# Understanding Object Exploration: The Role of Temperament Early in Life

**DOI:** 10.1111/infa.70109

**Published:** 2026-07-15

**Authors:** Kaylin E. Hill, Lauren Malachowski, Amy Work Needham, Kathryn L. Humphreys

**Affiliations:** ^1^ Department of Psychology University of Notre Dame Notre Dame Indiana USA; ^2^ Department of Psychology and Human Development Vanderbilt University Nashville Tennessee USA

**Keywords:** infancy, object exploration, surgency, temperament

## Abstract

Object exploration presents infants and young children with valuable opportunities for learning across domains. What factors motivate and constrain exploratory behavior? In the present studies, we highlight the infant's contributions to object exploration by testing links between parent‐reported temperament and observed exploratory behaviors. In two samples, Study 1: 24‐month‐olds (*N* = 35, 51% female; 88% White; 95% non‐Hispanic) and Study 2: 6‐month‐olds (*N* = 96, 50% female; 82% White; 89% non‐Hispanic), we assessed object exploration via coding of child behavior during two different lab‐based free play tasks. Across both samples, infants rated higher in temperamental surgency engaged in a more rapid pace of object exploration (i.e., moving quickly between objects). Additionally, cluster analyses indicated that infants characterized by high activity level, high positive affect, and high negative affect spent more time engaged in fine motor exploration (i.e., fingering and transferring) compared to infants characterized by average activity level, high positive affect, and low negative affect. These findings indicate a link between temperament and exploratory behavior in infancy and toddlerhood.

## 1 Introduction

The capacity to explore the environment is a crucial component of early childhood well‐being (Zeanah and Zeanah 2019), is an important reflection of early curiosity. Mounting evidence suggests that exploration in infancy facilitates learning across domains including language skills and academic achievement (Bornstein et al. 2013; Campos et al. 2000; Fry and Hale 1996; He et al. 2015; LeBarton and Iverson 2013; Wang et al. 2014; Yu et al. 2019; Zuccarini et al. 2017). The goal of the present study is to examine whether and to what degree motor exploration in infancy is related to infant internal characteristics or *temperament*. Object exploration refers to behaviors that young infants produce when an object is in or near their hands. Results of studies measuring these behaviors suggest that infants explore objects to learn about their characteristics and that infants use certain exploratory behaviors to extract certain kinds of information about the objects (e.g., Ruff 1984). The exploratory behaviors infants produce include looking, touching, and mouthing, and are often deployed in sequences that could help them connect information obtained across these different sensory modalities. For instance, Ruff et al. (1992) determined that mouthing followed by looking could be especially informative for infants' learning about an object. Similarly, Rochat (1989) claimed that transitions between looking and mouthing (in either direction) provided key information for infants to link information obtained via these two modalities, thus supporting a multi‐modal representation of the object.

It is common for researchers to place an object in an infant's hand(s) and let them engage in free exploration for a set period of time. The video of the infant's behavior can then be coded for looking (visual contact with the object), mouthing (oral contact with the object, including brushes against the lips), and manual contact (contacting the object with the hands and fingers, including fingertips sliding across the edges of the object). Other approaches include creating an exploration efficiency score that is a ratio of the number of play actions the infant engages in while exploring the toy divided by the total amount of time the infant spent holding the toy (e.g., Muentener et al. 2018). Object exploration behaviors develop rapidly across infancy, ranging from more rudimentary looking or touching behaviors to more sophisticated actions such as shaking, fingering, or rotating an object (Holland et al. 2023; Lobo and Galloway 2013; Ruff et al. 1992). Infants increasingly tailor their manual actions to the specific properties of objects, and this selectivity strengthens across the first year of life (Bourgeois et al. 2005; Palmer 1989). At the same time, interactions with peers and caregivers come to center more around toys and other objects (Bakeman and Adamson 1984; Schaffer 1984; Thurman and Corbetta 2017). During free play in the home environment, young children's interactions with toys and other objects are usually brief (median = 9.8 s) but accumulate to an estimated 60% of each waking hour, representing a substantial portion of a child's awake time (Herzberg et al. 2022). Together, changes across infancy mark a shift toward more intentional and coordinated engagement with the environment: across development, infants engage in more efficient visual exploration, more complex manual exploration, and more integrated visual–manual actions (Babik et al. 2022; Muentener et al. 2018).

From a developmental cascades perspective, these early exploratory acts—though seemingly mundane—serve as building blocks for later skills, as repeated experiences accumulate to affect development (Iverson 2021; Malachowski and Needham 2023; Masten and Cicchetti 2010). Early variability in motor skills (e.g., Franchak 2020) and object exploration (e.g., Iverson 2021) can set in motion downstream differences in learning opportunities and developmental outcomes. Small advances in exploratory skills may foster more robust communication with caregivers, access to more complex objects, and earlier language acquisition (Franchak 2020). For example, infants who engaged in more fine motor and oral exploration than their peers later scored higher on IQ and vocabulary tests, respectively (Zuccarini et al. 2018). Similarly, infants who explored a greater number of object components within a given time (i.e., demonstrated a faster exploration tempo) had larger vocabulary sizes and higher verbal comprehension by age 3 years (Muentener et al. 2018). Moreover, early individual differences in exploration appear to have remarkably long reach: infants who engaged in more rapid exploration at age five months had higher academic achievement at age 14 years, even after accounting for environmental factors (Bornstein et al. 2013). Bornstein and colleagues argue that exploration in early life initiates developmental cascades that influence cognitive growth and later achievement. Active explorers interact more with their surroundings, creating richer learning experiences—thus, infants help shape their own developmental trajectories (Bornstein et al. 2013).

Infant temperament may be one way to explain variation in infant exploration behaviors. Nonhuman animal models of temperament suggest that identifying constellations of traits can help explain individual differences in how organisms engage with their environments (Biro and Stamps 2008; Dingemanse and de Goede 2004; Quinn et al. 2009; Réale et al. 2007). Temperament in child development was initially defined as how children characteristically interact with their environment (Carey 1981; Thomas and Chess 1977). Building on this foundation, transactional models suggest that temperament represents both what an infant elicits from their environment and how they respond to it (Plomin and Daniels 1984; Sameroff 1975). These theories have been supported with empirical work that largely focuses on temperament interacting with environmental factors to predict behavioral outcomes (Paterson and Sanson 1999). In infancy, children engage with their environment namely through object exploration—with important implications for later skill acquisition. Recently, Altmann et al. (2025) developed the first caregiver‐report measure of infant curiosity and validated it by showing that curiosity scores correlate with temperament, positively with surgency and negatively with negative affect. This work represents an important step in connecting temperament to early exploratory motivation; however, it relies exclusively on parent‐reported measures and does not examine infants' exploratory behavior via careful frame‐by‐frame analysis in a standardized task. Accordingly, a direct link between temperament and how infants actually explore objects has not yet been established.

There is not universal agreement on how to characterize temperament; however, conceptual and empirical literature has focused on three distinct temperament dimensions: surgency, negative affectivity, and orienting/regulatory capacity (Gartstein et al. 2017; Putnam et al. 2014; Rothbart 2007; Rothbart and Bates 2006). Infants rated high on *surgency* (sometimes referred to as “exuberance”) have high levels of activity, high approach behaviors, and high positive affect (Putnam et al. 2014). Measures of surgency are generally stable through early childhood (Degnan et al. 2011). Infants rated high on *negative affectivity* have high levels of negative affect and a difficult time recovering from distress or excitement (Putnam et al. 2014). The final factor is orienting/regulatory capacity. Infants rated high on *orienting/regulatory capacity*, also referred to as *effortful control* across the literature, demonstrate focus for longer periods of time, lower levels of impulsivity, and higher levels of regulation in emotions and behaviors (Putnam et al. 2014). These temperament dimensions may differentially shape exploratory behavior: infants high in surgency may seek out novel stimulation and explore more rapidly; those high in negative affectivity may approach new objects more cautiously or withdraw more quickly; and those high in orienting/regulatory capacity may sustain attention and engage more persistently with objects (Rothbart and Bates 2006; Posner and Rothbart 2007). Children's behavior varies as a function of surgency, negative affectivity, and orienting/regulatory capacity, and according to constellations of temperament dimensions. For example, Gartstein et al. (2017) demonstrated that young infants (e.g., < 8 months of age) coalesce into three subgroups or clusters of individuals according to patterns observed across temperament dimensions. However, older infants (e.g., 9–12 months of age) coalesce into five subgroups of individuals across these dimensions, illustrating considerable developmental changes across the first year of infancy (Gartstein et al. 2017). Altogether, existing evidence suggests that object exploration may be one early behavior that is associated with temperament, with implications for linking variations in infant temperament to later developmental outcomes.

### 1.1 The Present Studies

The present studies are among the first to directly examine links between parent‐reported infant temperament and observed object exploration behavior. While recent work has established associations between temperament and curiosity via caregiver report (Altmann et al. 2025), whether and how temperament shapes the way infants physically engage with objects has not been examined. The overarching aim of the present work is to examine whether temperament shapes the way infants engage with objects during exploration. We address this question across two studies that differ in participant age, task structure, and the specific behaviors examined. Study 1 tests whether temperament relates to exploration tempo in 24‐month‐olds, an age at which exploratory behavior is relatively mature and purposive. Study 2 examines the same question in 6‐month‐olds using an extended naturalistic paradigm, with the additional aim of characterizing whether person‐centered profiles differentiate infants on more fine‐grained aspects of exploratory behavior, including fine motor engagement. The methodological and developmental differences between the two studies are features of the design: consistent findings across samples that differ in age, task context, and the range of behaviors examined would strengthen confidence that the observed associations between temperament and exploration reflect a robust phenomenon rather than an artifact of any particular context or developmental moment.

## 2 Study 1

First, we examined the associations between parent‐reported temperament and observed object exploration in a sample of infants aged 24‐months‐old at the time of the study. Temperament was measured using parent‐report and object exploration was measured in observing a 30‐s exploration session with a single object with 10 moveable subcomponents. Given the novel nature of our research aims, we considered the study exploratory and did not construct a priori hypotheses.

### 2.1 Methods

#### 2.1.1 Participants

Participants for Study 1 were 35 toddlers and their caregivers, who were a part of a longitudinal study (*N* = 50) examining early perceptual–motor experiences and tool‐use development. All procedures and recruitment methods were approved by the Institutional Review Board of Vanderbilt University Medical Center, and all participants provided informed consent prior to participating. The study was conducted in accordance with the ethical standards of the American Psychological Association. This study was originally designed to assess the role of early experiences on infants' object manipulation and tool use. To that end, half of the infants were assigned to receive object exploration opportunities at home between visits 1 and 2, and half were assigned to receive social interaction experiences between these two visits. Infants were recruited from the southern United States beginning in 2017 and followed longitudinally starting at 2.5 months and continuing at 3, 8.5, 14.5, and 23.5 months of age. However, the planned intervention was not delivered properly and we had attrition due to the COVID‐19 pandemic. We observed no statistically significant differences between the two groups in any of our outcome measures. The current analyses use data collected at the 23.5 months visit (parent responses to the ECBQ and infants' object exploration behaviors).

Of the original 50 participants, 14 participants were lost due to attrition (6 withdrew or were otherwise unavailable, 6 declined due to COVID‐19 concerns, 1 did not complete the questionnaires, and 1 was too fussy to complete the visit), and 1 was excluded from analyses for other reasons (their twin was a participant in the study). Attrition rates were comparable across conditions (experimental group: *n* = 8; control group: *n* = 7), and no demographic differences were observed between participants who completed and those who discontinued participation. The final sample (*n* = 35; 51% female) had a mean age of 24.14 months (SD = 1.44 months) and was predominantly White (88%), with 5% identifying as Asian, 2% as Black or African American, and 5% as more than one race; 5% identified as Latine. Most primary caregivers (94%) reported a bachelor's degree or higher.

#### 2.1.2 Procedure

**FIGURE 1:**
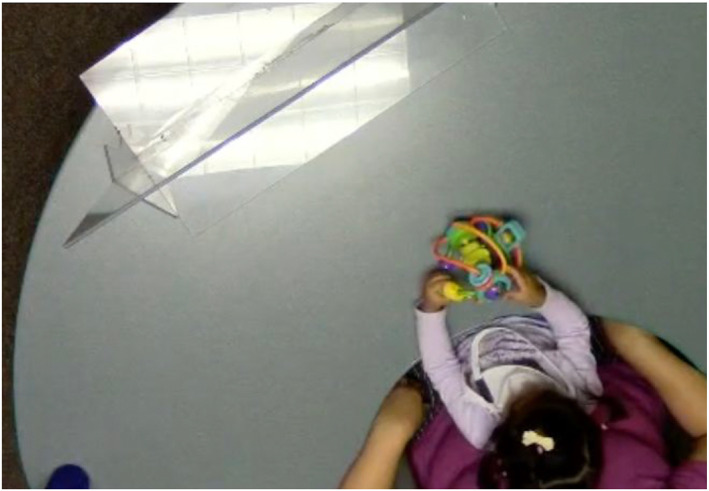
Overhead camera view of activity ball task. A clear plastic barrier was placed between the experimenter and the child.

The 24‐month visit consisted of several short behavioral tasks and a battery of questionnaires. In the present study, caregivers completed a questionnaire of infant temperament and infants completed an “Activity Ball” free play task. For this task, infants sat on their parent's lap at the edge of a rounded study table (see Figure 1). After a warm‐up task, the experimenter said, “Look what I have!” and presented the infant with an activity ball toy. After the presentation, the experimenter turned her back and pretended to be occupied by paperwork. Caregivers were asked before the session to refrain from interfering with their infant's actions—they were only permitted to briefly acknowledge the toy if their infant turned to show it to them. Infants were given 30 s to explore the activity ball however they wished before the experimenter turned around to retrieve it. The task was 30 s in duration as is common in the field of cognitive and perceptual development (Oakes and Tellinghuisen 1994; Palmer 1989; Ruff 1984). This task was filmed by four video cameras (overhead view, front view, and two side views) for later coding.

#### 2.1.3 Video Coding

The coding scheme used in the present study was based on the coding scheme described by Muentener et al. (2018) and Zuccarini et al. (2018). Prior to coding, we named each of the 10 moveable components of the Activity Ball toy (e.g., purple ring, blue caterpillar). A trained research assistant watched the video of the task and checked off each Activity Ball component that the toddler explored. Following criteria from previous research (Herzberg et al. 2022), the toddler had to move or displace the toy component for it to count as “explored.” Simply touching a component did not render it “explored.” Each component was only marked once, even if the infant manipulated it multiple times. This information was used to calculate the total number of components each infant contacted. The first author double‐coded 20% of the videos, with an average percent agreement with research assistants in the coding team (on the number of components contacted) of 96%. We used Datavyu to mark the onsets and offsets of infant exploration of the activity ball (Datavyu Team 2014). Several steps were taken to reduce potential coder bias. First, all videos were coded frame by frame with the camera view restricted to infants' hands. Second, temperament data were stored separately from the coding files and were not part of the workflow used for behavioral coding. Third, there were no a priori hypotheses, making specific temperament related biases in coding unlikely. Lastly, interrater reliability was high. Together, these factors make it highly improbable that knowledge of temperament influenced coding decisions.

#### 2.1.4 Specific Measures

##### 2.1.4.1 Parent‐Reported Temperament

Temperament was assessed using the Early Childhood Behavior Questionnaire—Very Short Form (ECBQ‐VSF; Putnam et al. 2014), completed by caregivers at child age 24‐months. The ECBQ‐VSF is a 36‐item measure. Items range from (1) *Never* to (7) *Always*. The total score for subscales is calculated as the mean of all items in the subscale; as such, subscales range from 1–7. Higher scores on each temperament scale indicate higher levels of that temperament dimension. Following standard scoring procedures, we computed three superordinate dimensions—Surgency, Negative Affectivity, and Orienting/Regulatory Capacity—from their corresponding subscales. Internal consistency for each dimension was estimated using Guttman's Lambda‐6 (Revelle 2022), which assesses the extent to which items measure a common construct. Reliability was acceptable in the present sample (Surgency = 0.77; Negative Affectivity = 0.74; Orienting/Regulatory Capacity = 0.83).

##### 2.1.4.2 Exploration Tempo

Exploration tempo was calculated by dividing the total number of unique activity ball components explored by the total number of seconds the infant spent engaged with the activity ball. For example, if the infant contacted 5 different components of the activity ball and spent a total of 25 s engaged with the object, a score of 0.2 was assigned.

#### 2.1.5 Sample Size Justification

The current analyses were developed post hoc as part of a dissertation project supplementary to a longitudinal study examining the development of tool‐use skills. The sample size reflects all eligible participants with behavioral exploration and temperament data at infant age 24 months. No a priori power analyses were conducted. Post hoc sensitivity analyses were calculated using a two‐tailed test, *α* = 0.05, *n* = 35, and Power = 0.80 in G*Power (Faul et al. 2009), and revealed the present study was powered at 80% to detect correlations *r* ≥ 0.33 as statistically significantly different from *r* = 0.00.

#### 2.1.6 Data Analysis

To reduce influence of outliers and account for non‐normal distribution of variables, we conducted Spearman's rank‐order correlations between exploration tempo and temperament factors from the ECBQ (surgency, negative affectivity, or orienting/regulatory capacity). We calculated 95% confidence intervals for these correlations using a bootstrapping method with 1000 repetitions using the RVAideMemoire package in R (Hervé 2018). The “very short” version of the ECBQ did not allow for the analysis of fine‐grained dimensions of temperament. De‐identified data and code are available at https://osf.io/paefs/.

### 2.2 Results

#### 2.2.1 Descriptives

On average, infants spent 26.18 s (SD = 7.00) engaged with the activity ball and contacted 3.80 components of the activity ball (SD = 1.95). Given the initial study design randomly assigned participants to experimental groups, we tested whether experimental conditions differed on exploration tempo. A Wilcoxon rank sum test indicated that the difference between experimental and control groups on exploration tempo was not statistically significant, *W* = 119, *p* = 0.270. Surgency and negative affectivity were not significantly correlated (95% CI [−0.30, 0.38]), surgency and orienting/regulatory capacity were not significantly correlated (95% CI [−0.16, 0.50]), and negative affectivity and orienting/regulatory capacity were significantly negatively correlated (95% CI [−0.73, −0.17]), such that infants who were rated higher in negative affectivity were likely to be rated lower in orienting/regulatory capacity. See Table 1 for a correlation table of all study variables.

**TABLE 1:** Study 1 correlation table.

	*M*	SD	1	2	3	4
1. Age (months)	24.14	1.44	—			
2. Exploration tempo	0.15	0.07	0.23	—		
3. Surgency	5.08	0.61	0.29	0.41*	—	
4. Negative affectivity	2.85	0.55	−0.02	−0.35*	0.04	—
5. Orienting/regulatory capacity	4.89	0.63	0.14	0.10	0.18	−0.48*

^*^ *p* < 0.05.

#### 2.2.2 Temperament and Exploration

**FIGURE 2:**
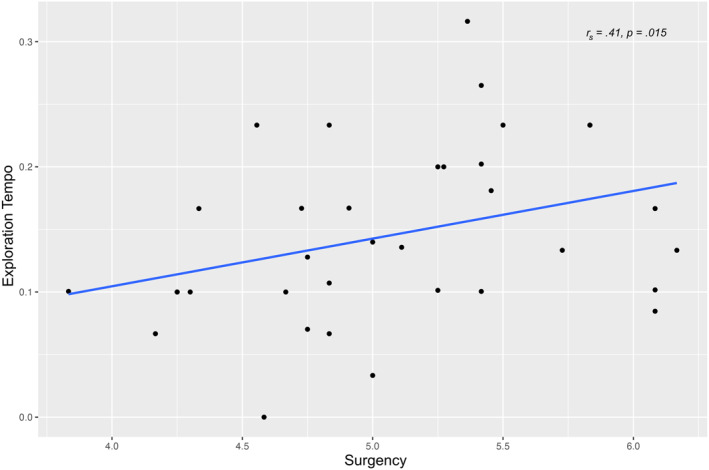
Scatterplot of surgency and exploration tempo. Plot of raw values and fitted regression line of the association between surgency and exploration tempo.

**FIGURE 3:**
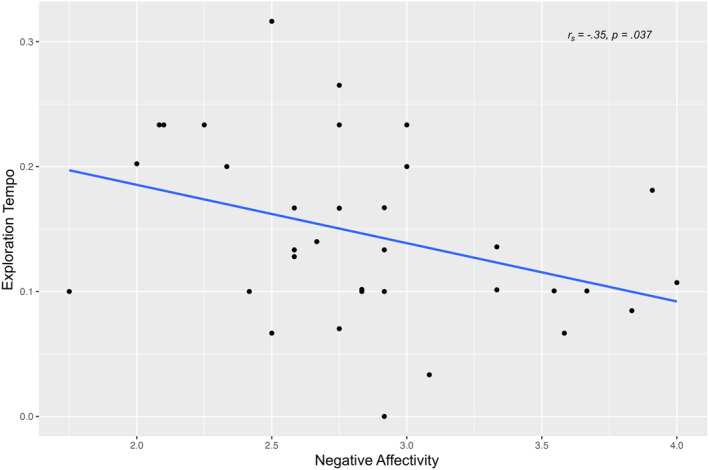
Scatterplot of negative affectivity and exploration tempo. Plot of raw values and fitted regression line of the association between negative affectivity and exploration tempo.

Spearman's rank‐order correlations between exploration tempo and each of the three temperament factors revealed a medium‐sized significant correlation between surgency and exploration tempo. Higher levels of surgency were associated with higher exploration tempo (95% CI [0.09, 0.63], *p* = 0.015; see Figure 2). Negative affectivity was also significantly correlated with exploration tempo, such that toddlers rated higher in negative affectivity demonstrated less rapid exploration of the activity ball (95% CI [−0.63, −0.01], *p* = 0.037; see Figure 3).

### 2.3 Study 1 Discussion

Toddlers whose parents rated them higher on surgency explored the activity ball at a faster tempo than those rated lower in surgency. Surgency is associated with reward sensitivity and responsiveness (Bunford et al. 2022; Gomez et al. 2016), which may manifest as more rapid exploration of a variety of objects in an effort to maximize the inherent reward of novelty. Highly surgent infants may also engage in a more rapid tempo of exploration because they are already experiencing positive affect. According to Fredrickson's Broaden and Build Theory (Fredrickson 1998), experiences of positive affect broaden behavioral repertoires, encourage discovery, and promote skill‐building (Seehagen et al. 2025; Stifter et al. 2020). Positive affect has been causally linked to enhanced cognitive performance (Blau and Klein 2010). An infant prone to display positive affect may be motivated to “discover,” as reflected in relatively rapid engagement with multiple objects or components of objects.

We also observed a significant association between negative affectivity and exploration tempo, such that toddlers rated higher in negative affect demonstrated a slower pace of exploration. This pattern is consistent with evidence that fearful temperament is associated with longer latencies to approach and engage with novel objects in infancy–infants higher in fearful distress reach more slowly for unfamiliar, high‐intensity toys, and this relationship strengthens across the first year as inhibited approach develops (Rothbart 1988). One possibility is that the novelty and social demands of the laboratory context constrained exploration disproportionately for toddlers higher in negative affect. Children higher in negative affectivity are characterized by greater reactivity to unfamiliar people, objects, and settings, and it is plausible that a structured laboratory task was experienced as more arousing or evaluative by more temperamentally reactive children, leading to more cautious and slower‐paced engagement. If so, the observed association between negative affectivity and exploration tempo may reflect, at least in part, context‐specific suppression of approach behavior rather than a stable dispositional tendency to explore slowly. This interpretation remains speculative, however, and longer observations across multiple contexts would be needed to evaluate whether the link between negative affectivity and exploration pace is robust or situationally moderated.

#### 2.3.1 Limitations

These data were collected within a study originally designed as an intervention. The intervention was not delivered as planned, and we observed no group differences in exploration tempo or in any other outcome. Because random assignment was preserved, systematic differences between conditions are unlikely. The design does mean, though, that infants may have varied in the activities and experiences they encountered before the exploration assessment, and we could not model that variability with these data.

The present study also focused on a single measure of object exploration: exploration tempo. There is not a single agreed upon measure or context for studying object exploration. Trials vary from 10 s to multiple minutes and the number of trials varies from 1 to 12 (Fidler et al. 2019; Muentener et al. 2018; Oakes and Tellinghuisen 1994; Palmer 1989; Rochat 1989; Ruff 1984; Ruff and Kohler 1978; Von Hofsten 1982). Similarly, assessments of object exploration range from observation of single behaviors to many, as well as in the specificity versus generality and in count versus speed metrics. The current study used a 30‐s task to examine toddler exploratory behavior while their locomotor and other behaviors were being restricted. This brief measurement allowed us to obtain data from most of our participants. Longer observations may be helpful for determining how stable and robust these associations are across early development, but these longer observations may not be well‐suited for this testing context. Moreover, exploration tempo offers one piece of information related to object exploration; however, additional measures of object exploration will provide greater understanding of the associations between dimensions of temperament and object exploration.

Findings regarding temperament and exploration may vary across cultural and demographic contexts. Both children's expression of temperament and caregivers' perceptions of those traits are shaped by cultural values (Super et al. 2008). For example, caregivers in the U.S. tend to value surgent characteristics such as high activity level more than caregivers in Israel (Klein and Ballantine 1991). Consequently, a highly surgent toddler in the U.S. may elicit encouragement and engagement, whereas a similarly active toddler in Israel may evoke efforts to dampen such behavior (Zentner and Shiner 2012). Future work should directly test whether cultural context moderates associations between temperament and exploratory behavior. Lastly, it should be noted that this study, and the next, provide post hoc sensitivity analyses for transparency. No a priori power analyses were conducted for these analyses, and all results should be interpreted with this in mind.

## 3 Study 2

Study 2 extends the aims of Study 1 in three conceptually important ways. First, whereas Study 1 focused on exploration tempo and examined temperament at the superordinate dimension level, Study 2 examines a broader suite of exploratory behaviors, including fine motor behaviors such as fingering and transferring that are theoretically linked to deeper object processing (Ruff 1984), and supplements the variable‐centered approach with a person‐centered analysis. Person‐centered analyses allow us to ask whether constellations of temperament dimensions, rather than individual dimensions examined in isolation, differentiate infants in their object engagement. Second, Study 2 was conducted during a naturalistic caregiver–infant free play session in which caregiver behavior was an active element of the environment. This allows us to examine whether the association between temperament and exploration tempo holds even when accounting for caregiver influence on object availability, providing a stronger test of whether observed effects reflect the infant's own behavioral tendencies. Third, the younger age of the Study 2 sample (6 vs. 24 months) allows us to evaluate whether temperament‐exploration associations are present early in the first year of life, before language and locomotion substantially reorganize infants' engagement with the environment. Examining these questions in a younger sample, in a naturalistic context, and with a broader behavioral coding scheme thus extends rather than merely replicates the aims of Study 1.

### 3.1 Methods

#### 3.1.1 Preregistration

Study aims and the basic data analysis plan for Study 2 were preregistered on OSF (https://osf.io/9ezng/). To build on the single measure of object exploration in Study 1, we planned to assess exploration across three variables: (1) exploration tempo, (2) manual exploration, and (3) oral exploration. We did not pre‐register a priori hypotheses for the planned analyses and thus Study 2 remains exploratory in nature. In addition to the basic correlational analysis plan described in the preregistration, we added a person‐centered approach to the data analysis plan. A person‐centered approach offers an alternative to the variable‐centered approach to temperament. The person‐centered approach offers additional information in examining associations between temperament and exploration in that it combines multiple dimensions into a single temperament “type” (e.g., “easygoing”). As such, we conducted person‐centered analyses, wherein temperament‐type‐based groups were compared in exploration behaviors.

#### 3.1.2 Participants

Participants were 96 infants and their birth mothers participating in a longitudinal pregnancy cohort study assessing early experiences, brain development, and behavior. There was not any overlap in participants across Studies 1 and 2. Pregnant individuals were recruited from the southern United States beginning in 2020. All procedures and recruitment methods were approved by the Institutional Review Board of Vanderbilt University Medical Center, and all participants provided informed consent prior to participating. The study was conducted in accordance with the ethical standards of the American Psychological Association. In the present study, we analyzed data from the follow‐up visit when infants were approximately 6‐months‐old–an age when research examining temperament in young infants has been successfully applied (i.e., 3–8 months of age; Gartstein et al. 2017). Mean infant age at this visit was 6.43 months (SD = 0.6 months). Infants were 50% female. Eighty‐two percent of participants were White; 5% were Black; 2% were Asian; 1% were Pacific Islander; and 6% identified as “Other” (e.g., multiracial). Eleven percent of participants identified as Latine. Income‐to‐needs ratios (INR) were calculated based on reported household income, number of people living in the home, and the 2020 U.S. Department of Housing and Urban Development's low‐income thresholds for U.S. residents reported on the government website (U.S. Department of Housing and Urban Development 2020). INRs ranged from 0.34 to 2.53 (*M* = 1.54, SD = 0.66), with INRs < 1.0 considered low‐income.

#### 3.1.3 Procedure

**FIGURE 4:**
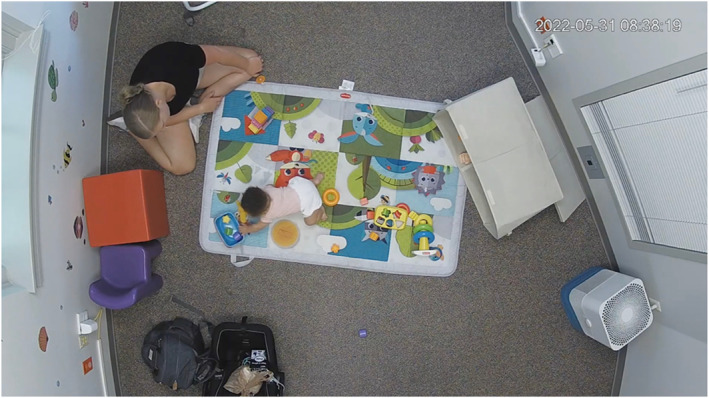
Overhead view of caregiver and child during the free play task.

The 6‐month lab visit included a filmed caregiver–child interaction task (“free play”) and a battery of questionnaires, including the temperament measure used in the present study. The free play task lasted 8 min and took place in a private lab room without the experimenter present. Caregivers were instructed, “Please play with your baby as you normally would at home. There are clean toys in the bucket, or you can use any that you may have brought with you.” The lab room contained a large playmat and a toybox full of age‐appropriate toys (see Figure 4). The playmat consisted of 7 object components and 14 toys were available in the toybox. There were no restrictions on the number of toys that could be accessed at any given time. The task was filmed at three angles: overhead and in two opposing corners of the room.

#### 3.1.4 Video Coding

Infant object exploration was measured via behavioral coding of the full 8‐min duration of the free play task. We used Datavyu software (version v1.3.8) to code the videos for infant object exploration frame by frame (Datavyu Team 2014). Following an existing coding scheme (Zuccarini et al. 2018), infant behaviors lasting ≥ 1 s were labeled with the specific exploratory behavior observed: hold (continuous manual contact with an object not involving another type of exploration), oral (object makes contact with the infant's lips, tongue, or mouth), rhythmic (repeated vigorous arm movement, either shaking an object or hitting it repeatedly), finger (scanning, scratching, or pinching an object's surface with fingertips), or transfer (moving an object from one hand to the other). We originally had a sixth behavior category–rotate (holding an object in view and rotating it, as if to see the other side). However, instances of rotating were not clear from the video footage, so we incorporated this behavior into the most closely related “hold” coding category. We recorded two behaviors (e.g., hold and oral) if the infants' two hands were simultaneously engaging in two different behaviors; there were 300 total segments of two behavior codes listed, which was approximately 4% of all segments. These instances were later labeled as “multiple” for analysis.

A new cell was created in Datavyu for each type of behavior. When a new behavior became clear, the coder offset the previous behavior in Datavyu and onset a new one. Any segment in which the coder's view of the infants' hands was obstructed was labeled “no code.” In addition to exploratory behavior type, we recorded the specific object or objects being explored. Names of objects were pre‐determined to ensure consistency within each video and between raters. Multiple objects were listed if the infant was engaging simultaneously with more than one object.

Objects were defined as any object that could be lifted and displaced by the infant; however, these did not include clothing on the infant or caregivers' bodies, shoes, or pacifiers. There was only one available toy (a popup toy) of the 14 provided that did not fit our definition of “object” because of its large size and difficulty to manipulate. To avoid a situation where infants who played with the popup toy received lower exploration scores due to their focus on the popup toy, we labeled segments of popup exploration as “no code” (i.e., they were removed from “codable” segments of the video).

Primary coding of each video was completed either by the first author or a trained research assistant. For reliability, a randomly selected segment of each video (comprising 25% of the total video) was independently coded by both the first author and a trained research assistant. Total durations of each type of exploratory behavior within the double‐coded segments were calculated and compared between raters. Intra‐class correlations (ICCs) between the two raters were 0.99 for all object contact, 0.98 for hold, 0.98 for oral, 0.91 for rhythmic, 0.84 for finger, and 0.84 for transfer. Based on widely accepted benchmark guidelines, reliability for each behavior was considered either “good” or “excellent” (Bobak et al. 2018). Disagreements between raters were discussed after reliability coding, and a final column was created to reflect any changes based on discussion.

Because caregivers could use any number of toys in the toy box, we also marked the videos for each unique object that was available to the infant throughout the free play session. Two trained research assistants watched the videos and recorded the name of each new object made available to the infant. Availability was defined as being within reach of the infant for at least 1 s and could result either from caregiver behavior (e.g., caregiver placing an object within the infant's reach) or infant behavior (e.g., infant rolling or crawling). Each video was double‐coded for object availability, with 96% agreement.

The final coding columns were exported from Datavyu and scored in *R* Version 4.2.0 (R Core Team 2020). Total durations of each behavior (hold, oral, finger, rhythmic, transfer) as well as the number of unique objects contacted throughout the free play session were calculated for each infant. Of note, the number of objects available was not considered when calculating the number of unique objects contacted by infants. Instead, we calculated the number of object contacts and the number of objects available separately to maintain specificity in examining these associations.

Several steps were taken to reduce potential coder bias. First, all videos were coded frame by frame. Second, temperament data were stored separately from the coding files and were not part of the workflow used for behavioral coding. Third, there were no a priori hypotheses, making specific temperament related biases in coding unlikely. Lastly, interrater reliability was high across exploration measures. Together, these factors make it highly improbable that knowledge of temperament influenced coding decisions.

#### 3.1.5 Specific Measures

##### 3.1.5.1 Parent‐Reported Temperament

Temperament was measured using the Infant Behavior Questionnaire–Revised Short Form (IBQ‐R‐SF; Gartstein and Rothbart 2003; Putnam et al. 2014; Rothbart 1981), completed by caregivers at infant age 6‐months. The IBQ‐R‐SF is the infant version of the ECBQ‐VSF used in Study 1. Although the ECBQ‐VSF and IBQ‐R‐SF differ in item number and specific subdimensions, both are derived from the same Rothbart temperament framework and yield comparable higher‐order dimensions. The IBQ‐R‐SF is a 91‐item measure, wherein items range from (1) *Never* to (7) *Always*. On the Short version, 14 subdimensions of temperament can be scored as well as 3 superordinate dimensions. In alignment with measurement in Study 1 and the preregistration analytic plan of the current study, we used the 3 superordinate dimensions in the planned variable‐centered analyses. Specifically, Surgency was calculated by including the items that constitute the subscales of approach, vocal reactivity, high‐intensity pleasure, smiling and laughter, activity level, and perceptual sensitivity; Negative Affectivity was calculated by including the items that constitute the subscales of sadness, distress to limitation, fear, and falling reactivity. Orienting/Regulatory Capacity was calculated by including the items that constitute the subscales of low‐intensity pleasure, cuddliness, duration of orienting, and soothability. The total score for each dimension was calculated as the mean of all items in the dimension; as such, subscales range from 1–7. Higher scores on each temperament dimension indicate higher levels of that temperament dimension. The internal consistency of these 3 dimensions were excellent in the present sample (surgency: G6 = 0.97; negative affectivity: G6 = 0.94; orienting/regulatory capacity: G6 = 0.93).

##### 3.1.5.2 Gross Motor Skill

Given the relevance of motor skills for object exploration behavior, infant gross motor skill was included as a covariate in analyses. Gross motor skill was assessed via the Gross Motor subscale of the Ages and Stages Questionnaire (ASQ; Squires and Bricker 2009), completed by caregivers regarding at infant age 6‐months. The ASQ Gross Motor subscale contains six items. Items range from (0) *Not Yet* to (10) *Yes* for each queried skill. The total AASQ Gross Motor Skill score is calculated as a sum of the six items; as such, the subscale ranges from 0 to 60 and higher scores indicate more motor skills.

##### 3.1.5.3 Durations of Individual Exploration Behaviors

Durations of each coded exploration behavior (hold, oral, rhythmic, finger, transfer) were calculated as the total number of seconds the infant spent engaged in that behavior. All durations were analyzed as proportions of codable video (i.e., the total length of the free play task, excluding times when the coder's view of the infant was obstructed) to account for slight differences in task time between infants. Specifically, the duration of codable video was calculated as the total length of the task minus the total duration of “no code” periods.

##### 3.1.5.4 Duration of Fine Motor Exploration

Fine motor exploration was coded separately from object exploration due to the unique nature of these exploratory behaviors (Rachwani et al. 2020; Taylor et al. 2024). Fine Motor duration was calculated as the total number of seconds the infant spent engaging in either fingering or transferring of objects (Wolff 2020).

##### 3.1.5.5 Duration of Overall Exploration

Duration of overall exploration was calculated as the total number of seconds the infant spent engaged in any type of exploration with an object.

##### 3.1.5.6 Exploration Tempo

Exploration tempo, referred to as “efficiency” in previous work, was calculated by dividing the total number of unique objects contacted by the infant by the total duration of time the infant spent engaged in any type of object exploration (Muentener et al. 2018). This total duration was not a proportion of codable video because we wanted to know how many objects were contacted in the time we observed the infant engaged in exploration. If an infant contacted three objects throughout the full free play session and spent a total of 240 s engaging in object exploration behaviors, we assigned the infant a score of 0.01 (3/240). This variable is a unique combination of the overall *breadth* of exploration as well as the pace at which infants explore.

##### 3.1.5.7 Number of Available Objects

The number of available objects was defined as the total number of unique objects available to the infant throughout the free play session. A given object was counted only once, even if it was taken away and later returned to the infant. Of note, the popup toy was not counted in the number of available objects given that periods in contact with this toy were coded as “no code”; please see below.

#### 3.1.6 Sample Size Justification

The current analyses were developed post hoc as part of a dissertation project supplementary to a longitudinal pregnancy cohort study. The sample size reflects all eligible participants with behavioral exploration and temperament data at infant age 6 months at the time analyses were conducted. No a priori power analyses were conducted. For variable‐centered analyses, post hoc sensitivity analyses were calculated using two‐tailed test, *α* = 0.05, *n* = 96, and Power = 0.80 in G*Power (Faul et al. 2009), and revealed the present study was powered at 80% to detect correlations *r* ≥ 0.20 as statistically significantly different *from r* = 0.00. Post hoc sensitivity analysis approaches are not firmly established for cluster analysis (Dalmaijer et al. 2022), the person‐centered approach used in the present study. However, current guidelines established through simulation studies suggest samples *n* ≥ 20 per subgroup are adequately powered.

#### 3.1.7 Data Analysis

##### 3.1.7.1 Variable‐Centered Approach

As in Study 1, we conducted Spearman's rank‐order correlations between exploration measures (oral, fine motor, and tempo) and temperament factors from the IBQ (surgency, negative affectivity, and orienting/regulatory capacity). We calculated 95% confidence intervals for these correlations using a bootstrapping method with 1000 repetitions using the RVAideMemoire package in R (Hervé 2018).

##### 3.1.7.2 Person‐Centered Approach

As an exploratory analysis, we conducted a *k*‐means cluster analysis, wherein infants were placed into cluster groups based on scores on each of the 14 individual IBQ dimensions (activity level, distress to limitations, approach, fear, duration of orienting, smiling and laughter, vocal reactivity, sadness, perceptual sensitivity, high intensity pleasure, low intensity pleasure, cuddliness, soothability, falling reactivity/rate of recovery from distress). *K*‐means cluster analysis is a person‐centered, data‐driven approach wherein all information (in this case, the 14 temperament dimensions) are used to create the optimal number of groups (or clusters of individuals) that minimize intragroup variation and maximize intergroup variation (Hair et al. 2019). Cluster analyses were conducted in R using the *k‐means* function in the *stats* package (R Core Team 2020).

The number of identified clusters were then compared on object exploration metrics (oral, fine motor, and tempo) using Dunn's tests computed using the *dunn_test* function in the *rstatix* package (Kassambara 2023), a multiple‐comparison technique that compares the mean rank of different groups. De‐identified data and code are available at https://osf.io/paefs/.

### 3.2 Results

#### 3.2.1 Descriptives

Mean temperament ratings were 4.90 (SD = 0.69) for surgency, 3.18 (SD = 0.72) for negative affectivity, and 5.30 (SD = 0.59) for orienting/regulatory capacity. On average, infants spent 62% of the time engaged with objects. Infants spent 45% of the time holding, 9% of the time mouthing, 4% of the time engaged in rhythmic play, and 3% of the time engaged in fine motor exploration (2% fingering and 1% transferring). Surgency and negative affectivity were not significantly correlated, 95% CI [−0.14, 0.26]; surgency and orienting/regulatory capacity were significantly positively correlated (95% CI [0.18, 0.59]) such that infants rated higher in surgency were also rated higher in orienting/regulatory capacity; and negative affectivity and orienting/regulatory capacity were significantly negatively correlated (95% CI [−0.52, −0.16]), such that infants rated higher in negative affectivity were rated lower in orienting/regulatory capacity. See Table 2 for a correlation table of all study variables.

**TABLE 2:** Study 2 correlation table.

	*M*	SD	1	2	3	4	5	6	7	8	9
1. Age (weeks)	27.95	2.64	—								
2. Gross motor skill	47.42	9.59	0.19	—							
3. Duration of overall exploration	0.62	0.20	0.09	−0.11	—						
4. Duration of oral exploration	0.09	0.09	0.07	0.02	0.32*	—					
5. Duration of fine motor exploration	0.03	0.03	0.17	0.14	0.23*	−0.12	—				
6. Exploration tempo	0.02	0.02	0.15	0.23*	−0.54*	−0.35*	−0.10	—			
7. Number of available objects	7.02	3.82	0.23*	0.24*	−0.02	−0.09	0.01	0.70*	—		
8. Surgency	4.90	0.69	−0.20	0.21*	−0.22*	−0.07	0.03	0.27*	0.12	—	
9. Negative affectivity	3.18	0.72	0.16	−0.08	0.05	−0.09	0.19	0.07	0.16	0.07	—
10. Orienting/regulatory capacity	5.30	0.59	−0.19	0.00	−0.06	−0.13	−0.10	0.17	0.05	0.40*	−0.35*

^*^ *p* < 0.05.

#### 3.2.2 Variable‐Centered Approach

**FIGURE 5:**
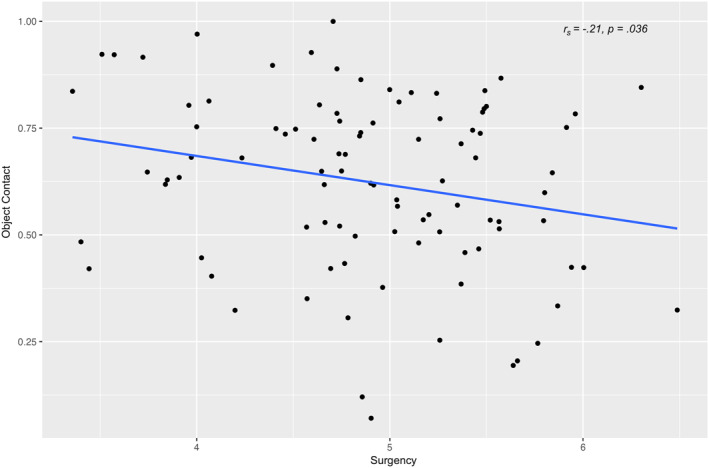
Scatterplot of surgency and object contact. Plot of raw values and fitted regression line of the association between surgency and object contact.

**FIGURE 6:**
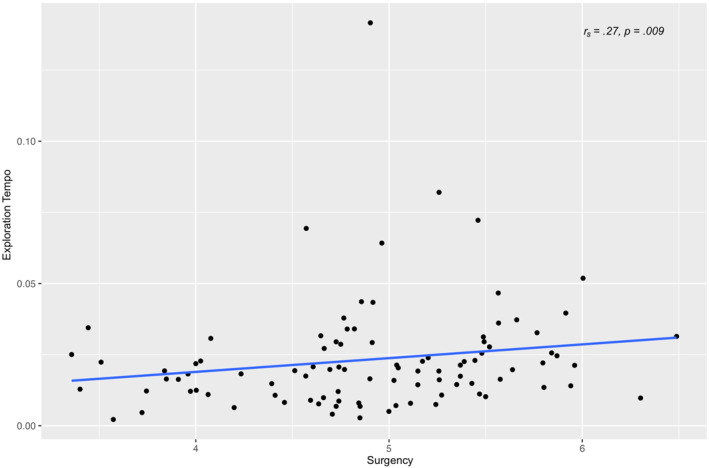
Scatterplot of surgency and exploration tempo. Plot of raw values and fitted regression line of the association between surgency and exploration tempo. The correlation remained significant when removing a participant with an extreme score on exploration tempo (*r*_s_ = 0.27, 95% CI [0.06, 0.44], *p* = 0.009).

Higher infant surgency was associated with significantly *less* overall time interacting with objects (*r*_s_ = −0.21, 95% CI [−0.42, −0.01], *p* = 0.036) and *faster* exploration tempo (*r*_*s*_ = 0.27, 95% CI [0.08, 0.42], *p* = 0.009).^1^ See Figures 5 and 6. The number of objects available to the infant was highly correlated with exploration tempo (*r*_s_ = 0.70, 95% CI [0.57, 0.80], *p* < 0.001) such that as the number of objects available increased, so did infant exploration tempo. To account for the impact of parental behavior in making objects available, we used a partial Spearman's correlation to examine the association between exploration tempo and surgency controlling for objects available. The positive correlation between tempo and surgency remained significant when accounting for the number of objects available to the infant (*r*_s_ = 0.20, *p* = 0.040). The positive correlation between surgency and tempo also remained significant when accounting for infants' gross motor skill (*r*_s_ = 0.25, *p* = 0.009). Effect sizes between negative affectivity and orienting/regulatory capacity and metrics of exploration were small and not statistically significant.

#### 3.2.3 Person‐Centered Approach

The silhouette method supported a 3‐cluster solution (*k* = 3). Cluster 1 (“High Positive/Low Negative”) was characterized by high levels of positive affect, low levels of negative affect, and average activity levels. Cluster 2 (“Active/High Positive/High Negative”) was characterized by high levels of both positive and negative affect as well as high levels of activity. Cluster 3 (“Low Active/Low Positive/Low Negative”) was characterized by below‐average levels on nearly all 14 subfactors. See Supporting Information S1: Table S1 for the mean values of each temperament factor by cluster and Supporting Information S1: Figure S1 for a boxplot depicting fine motor exploration by cluster, both available in the supplement.

A Kruskal–Wallis rank sum test indicated at least one significant difference between clusters on fine motor exploration, *χ* ^2^ = 7.17, *p* = 0.028. A Dunn's test (*dunn_test.R*) of multiple comparisons indicated that infants in Cluster 2 (“Active/High Positive/High Negative”) spent twice as much time engaged in fine motor exploration (i.e., fingering and transferring; *M* = 0.04 or 4% of the time, SD = 0.05) than did infants in Cluster 1 (“High Positive/Low Negative”; *M* = 0.02 or 2% of the time, SD = 0.03), *z* = 2.68, *p* = 0.022. Dunn's test ranks all values across clusters and compares mean ranks between groups; thus, it is insensitive to the outliers observed in Cluster 2 (Supporting Information S1: Figure S1).

### 3.3 Study 2 Discussion

#### 3.3.1 Object Exploration

We observed that infants spent an average of 5 min engaged with objects across the 8‐min task. This observation was further specified as approximately 3.6 min holding objects, 43 s mouthing objects, 19 s in rhythmic play, and 15 s in fine motor exploration. These durations are commonly observed in experimental studies designed to assess infant and toddler object exploration from the perspective of cognitive, perceptual, and motor development. Foundational lab‐based research in this field used 30‐s trials in their studies (Oakes and Tellinghuisen 1994; Palmer 1989; Ruff 1984). Moreover, recent research using naturalistic methods to observe infants' natural play at home found that the vast majority of interactions with objects are ≤ 30 s (Herzberg et al. 2022).

#### 3.3.2 Exploration Tempo

Consistent with Study 1, we found a weak but statistically significant rank‐order correlation between surgency and exploration tempo, such that infants rated higher on surgency engaged in a more rapid tempo of exploration. This association held even when covarying for infant gross motor skill, a developmental domain thought to provide a foundation for new learning opportunities (Adolph and Hoch 2020). The consistency of findings across the two studies is noteworthy, particularly given their methodological differences. Consistent findings across groups of 6‐month‐olds and 24‐month‐olds suggest that temperament and exploration behaviors seem to be related across early development. The nature of the object exploration task differed substantially between studies. In Study 1, the caregiver was asked to limit interaction with their child while the child explored a single object. In Study 2, caregivers were free to interact with the infant and the box of toys as they pleased. These important methodological differences increase our confidence in the link between the temperamental dimension of surgency and exploration tempo that moves beyond specific context. However, further research is needed to clarify possible links between temperament and object exploration.

#### 3.3.3 Fine Motor Exploration

The person‐centered clusters observed in the present study roughly mapped onto the 3 clusters found previously in a similar age group (Gartstein et al. 2017). We found that infants in the “Active/High Positive/High Negative” cluster (Cluster 2) engaged in more fine motor exploration than infants in the “High Positive/Low Negative” (Cluster 1) category. Compared to infants in Cluster 1, infants in Cluster 2 were substantially more active. The two fine motor behaviors assessed—fingering and transferring—require relatively complex motor movement and a higher level of attentional focus compared to more basic actions such as mouthing and holding. Specifically, transferring requires the infant to simultaneously coordinate the movement of both hands to “pass” an object from one hand to another. Fingering (i.e., running fingers along the surface of an object) typically requires the infant to hold an object steady with one hand while coordinating finger movement with the other. Active infants may simply have more energy to devote to such complex movements compared to less active infants.

Clusters 1 and 2 also differed in levels of negative affectivity: infants in Cluster 1 were reported to display low levels of negative affect, whereas those in Cluster 2 showed higher levels. Infants in Cluster 1 may have been content to engage in more basic behaviors (e.g., mouthing, holding, rhythmic) for longer periods of time. Additionally, infants with higher levels of negative affect may be overall more *sensitive* to stimulation from the environment, which may also make them more likely to engage in behaviors that provide more detailed information about objects. For example, infants tend to use fingering strategies to gain information about object texture and transferring to gain information about object weight (Ruff 1984).

Fine motor exploration at 6 months of age has been linked to 24‐month cognitive performance on a standardized test (Zuccarini et al. 2017). Fingering (or “haptic scanning”) may be especially important for infants' object perception (Gerhard et al. 2021). Thus, the combination of high activity level, high positive affectivity, and high negative affectivity may motivate an exploratory behavior i.e. closely tied to cognitive development.

#### 3.3.4 Comparing Variable‐ and Person‐Centered Approaches

The variable and person‐centered approaches to temperament provide unique insights into human behavior. Based on findings from Study 2, surgency may be more useful for understanding broader characteristics of exploration (e.g., tempo and total duration of object contact) but less so for understanding more fine‐grained behaviors like fine motor exploration. Likewise, person‐centered temperament clusters may be especially useful for understanding variation in fine‐grained behaviors.

#### 3.3.5 Limitations

Importantly, the present results are based on caregiver‐report of child temperament, and not observed temperament within the 8‐min task. As noted in the Study 1 Discussion, caregiver‐reported temperament may be more generalizable across experiences, settings, and time; however, this measurement method does not reflect direct observation of child behavior. Another limitation of Study 2 is that we did not directly code the caregiver's behavior, and thus whether and how caregiver's behaviors may relate to an infant's expression of temperament and how these behaviors shape the infant's exploration are not examined. Additionally, although the free play task allows us to capture naturalistic interaction between infants, caregivers, and objects, seated play with toys may occur relatively infrequently in day‐to‐day life. While interaction play tasks are common in coding infant behaviors (e.g., Zuccarini et al. 2018), it is important to note the possible effects of caregiver presence and interaction in interpreting the findings of these studies.

Relatedly, we did not directly code postural position in the present study, and thus it remains unclear if and to what degree infant exploration behaviors may differ according to body position. Infant posture, which was allowed to vary naturally during the free play task, may contribute to individual differences in exploration patterns, as postural control and object exploration are known to be linked (Gibson 1988; Rochat and Goubet 1995; Soska and Adolph 2014). For example, previous experimental work demonstrated that infants placed in supported sitting positions were more likely to engage in manual and oral exploration compared to lying positions (Mlincek et al. 2022; Soska and Adolph 2014). There are also observed costs to infant placement in supported sitting positions (e.g., using infant seating devices), lower language exposure (Malachowski et al. 2023), which may indicate fewer meaningful caregiver–infant interactions and opportunities for learning. While posture was not systematically coded in the present study, caregivers typically supported their infants in positions conducive to object manipulation. Future research could benefit from explicitly examining how postural variations might correlate or interact with temperamental differences to influence exploratory behavior. Finally, while a strength of the present study was the use of both variable‐centered and person‐centered analyses, power and sensitivity analysis approaches are not firmly established for cluster analysis. Findings from this analysis should be interpreted with this in mind.

## 4 General Discussion

### 4.1 Summary of Findings

In Study 1 (*N* = 35), we assessed object exploration via a structured free exploration task with a group of 24‐month‐old infants. In Study 2 (*N* = 96), we assessed object exploration via an unstructured infant–parent free play task with a sample of 6‐month‐old infants. Across both studies, the temperamental dimension of surgency–characterized by high levels of activity, approach, and positive affectivity–was positively and significantly associated with exploration tempo. Consistent findings between Studies 1 and 2 despite methodological differences increase our confidence in the observed effect. These findings also extend the recent work by Altmann et al. (2025), who established associations between temperament and parent‐reported infant curiosity, including a positive link between surgency and curiosity scores. The present studies complement that work by demonstrating that surgency relates not only to how caregivers characterize their infants' curiosity, but to how infants actually behave during object exploration. That this association holds across two samples, two age groups, and two methodologically distinct tasks suggests it is not an artifact of shared method variance between caregiver‐reported temperament and caregiver‐reported curiosity, but rather reflects a meaningful relationship between temperament and observed exploratory behavior. In Study 2, we also conducted cluster analyses based on the 14 subfactors present in the short form of the Revised Infant Behavior Questionnaire. While fine motor exploration was rare overall, infants rated higher in activity, positive affect, and negative affect (“Active/High Positive/High Negative”) engaged in twice as much fine motor exploration (fingering and transferring) as did infants with lower levels of activity and negative affect.

### 4.2 Surgency and Exploration Tempo

What are the potential implications of the observed association between surgency and object exploration? On the one hand, rapid exploration of objects may provide more opportunities for learning. For example, a highly surgent infant may make contact with more objects over the course of a given day compared to a less surgent infant, perhaps due to increased processing speed (Colombo et al. 1991). Over time, these moments of contact with objects may accumulate to produce meaningful increases in learning opportunities (e.g., more opportunities to hear object labels from caregivers). This idea is supported by evidence that rapid exploration tempo is longitudinally associated with higher IQ and educational attainment (Bornstein et al. 2013; Muentener et al. 2018). On the other hand, components of surgency have been linked to impulsivity (Fox et al. 2001; Pfeifer et al. 2002; Stifter et al. 2008). Highly‐surgent infants' rapid tempo of exploration may reflect this impulsivity, such that they tend to act on immediate impulses to interact with or explore other objects in their field of vision, even if they had just begun exploration of the first object. A rapid tempo of exploration may reduce the likelihood that infants engage in exploratory strategies (e.g., fingering, transferring) that require extended focus on a single object. One potential consequence is that infants may “miss out” on a depth of learning made possible by extended focus on an object.

### 4.3 Interactions Between Temperament and External Influences

The various factors related to object exploration may be best understood in the larger context of Bronfenbrenner's ecological systems theory (Bronfenbrenner 1979), in which personal characteristics (i.e., temperament, sex) and varying levels of external factors (i.e., immediate context, family context, neighborhood context, etc.) interact to shape behavior and development. Just as external influences and personal characteristics may directly shape a child's behavior, characteristics of the child (e.g., highly active) may elicit particular responses from caregivers that, in turn, shape infant behavior and further reinforce personal characteristics (as in a developmental cascades model; Iverson 2021). For example, sustained attention is positively associated to object exploration in early development (Ruff and Rothbart 2001), and there is work suggesting that this association may be both the result of (1) joint attention with caregivers supporting the development of infant sustained attention (Yu and Smith 2016) and (2) infant object exploration eliciting joint attention and reciprocal object exploration with caregivers (Bridgett et al. 2011; Tamis‐LeMonda et al. 2013). Similarly, advances in physical skills (e.g., sitting independently) are associated with changes how caregivers and young children “co‐construct” interactions and play (Schneider et al. 2023).

One challenge for future research is to tease apart the relative contributions of temperament and contextual influences on infant exploratory behavior. While the present studies demonstrated support for associations between child temperament and exploration, previous studies also demonstrate support for contextual influences. For example, infants who participated in training that allowed them to obtain objects actively (i.e., using “sticky mittens”), versus passive viewing of objects held by a parent, at age 3 months were reported by parents to have higher levels of attention focusing at child age 15 months in comparison to the control groups without “sticky mittens” training (Libertus et al. 2016). This experiment suggests motor skill training opportunities may influence the development of at least one temperament dimension (orienting/regulatory capacity; Libertus et al. 2016). In the present studies, Study 2 demonstrated that the number of objects available to the infant was a strong and significant predictor of exploration tempo, highlighting the influence of contextual factors on exploratory behavior. However, we did not find that temperament interacted with this particular contextual factor. The number of unique objects available to the infant may have been a crude proxy for caregiver behavior, so future studies should seek to capture more nuanced aspects of caregiver behavior during infant play with objects. Further, neither study assessed infant familiarity with the objects coded for exploration. Future studies should examine if, and to what degree, object familiarity relates to exploration behavior as well as the observed associations between exploration and temperament. Additionally, the present studies used caregiver‐report of infant and toddler temperament. Laboratory observations are limited by their brief duration and single‐context nature, which constrains their generalizability to broader temperamental dispositions across experiences, settings, and time (Rothbart et al. 2009). However, both caregiver‐report and laboratory observation methods of infant temperament measurement are prospectively associated with child functioning (Morales et al. 2022). Future research could consider teasing apart the value of each assessment method in relation to understanding object exploration behaviors.

### 4.4 Implications for Development

From a developmental cascades perspective, different “styles” of object exploration have meaningful implications for developmental trajectories. When accumulated across minutes, hours, days, and weeks in an infant's life, small differences in the tempo of object exploration may result in widely different opportunities for learning about objects (Franchak 2020). Infants who explore more rapidly may also elicit more frequent feedback from caregivers, resulting in more frequent opportunities for learning language, for example.

### 4.5 The Active Infant

The findings of the present studies emphasize the role of the child as an active agent in their environments. This sharply contrasts with theories, both historical and current, that emphasize or *over*‐emphasize the role of the environment. The Tabula Rasa (“blank slate”) theory of child development, first posited by John Locke in the 18th century and popularized by B. F. Skinner in the 20th century, positions the infant as a largely passive receiver of stimulation. Today, this line of thinking continues in what some authors call “radical environmentalism,” or the tendency to over‐emphasize the influence of the environment when explaining or predicting behavior (Lilienfeld 2010; Lykken 1991). According to Lilienfeld (2010), the problem with this stance is that an exclusive focus on experiences can cloud more nuanced perspectives on the complex interplay of the individual and the environment throughout development. Thus, if our goal is to accurately explain and predict human behavior, we must not forget the crucial role of individual difference factors, including temperament.

### 4.6 Conclusion

The present studies merge two broad domains of infant development not previously considered together: the motor domain (object exploration) and the affective domain (temperament). Findings from the present studies indicate a link between parent‐reported temperament and components of object exploration in infancy and toddlerhood and support a developmental cascades framework in which developments in one area are intricately connected to developments in other areas (Masten and Cicchetti 2010; Oakes and Rakison 2019). In the case of the present studies, early expressions of temperament may play a role in the development of behavioral patterns related to object play. It is important to note that the observed effect sizes were modest and the possibility of Type I error cannot be ruled out without replication in carefully planned studies with a priori power analyses justifying the sample size. The present findings should be understood as establishing an initial empirical link between temperament and exploration. Future research might consider how temperament shapes other important sources of cognitive stimulation in infancy (e.g., language input, caregiver scaffolding). Overall, our findings indicate that early learning experiences may depend on the specific behavioral styles and sets of preferences that infants bring into the world. This understanding points to the complexity of early development and the importance of examining both contextual and within‐person factors when seeking to explain and predict behavior.

## Author Contributions

**Kaylin E. Hill:** writing – review and editing, formal analysis, writing – original draft. **Lauren Malachowski:** conceptualization, investigation, writing – original draft, methodology, visualization, formal analysis, data curation. **Amy Work Needham:** conceptualization, funding acquisition, writing – review and editing, supervision. **Kathryn L. Humphreys:** conceptualization, funding acquisition, writing – review and editing, supervision.

## Funding

This research was supported by the Jacobs Foundation Early Career Research Fellowship 2017‐1261‐05 (Kathryn L. Humphreys); National Science Foundation CAREER Award 2042285 (Kathryn L. Humphreys) and DLS Award 1651075 (Amy Work Needham); Brain and Behavior Research Foundation John and Polly Sparks Foundation Investigator Award 29593 (Kathryn L. Humphreys); Vanderbilt Institute for Clinical and Translational Research Grant VR53419 (Kathryn L. Humphreys); Vanderbilt Strong Grant (Kathryn L. Humphreys); Vanderbilt Kennedy Center Grant (Kathryn L. Humphreys); and National Institutes of Health (T32‐MH18921 and K23MH131753, Kaylin E. Hill).

## Ethics Statement

All procedures performed with human subjects were in accordance with ethical standards and approved by the Vanderbilt University Medical Center Institutional Review Board.

## Consent

Informed consent was obtained from all parents of the infants included in both studies.

## Conflicts of Interest

The authors declare no conflicts of interest.

## Supporting information


Supporting Information S1

## Data Availability

The data that support the main findings of this study are openly available at https://osf.io/paefs/.

## Endnotes

^1^
We calculated exploration tempo accounting for codable time. That is, we revised the original calculation from *tempo = number of distinct objects contacted/time spent in contact with objects* to *tempo = number of distinct objects contacted/time spent in contact with objects/total codable time*. The correlation between revised exploration tempo, accounting for codable time, and surgency was consistently observed, *r*_s_ = 0.27, *p* = 0.007.

## References

[infa70109-bib-0001] Adolph, K. E. , and J. E. Hoch . 2020. “The Importance of Motor Skills for Development.” In Nestle Nutrition Institute Workshop Series 95: 136–144. 10.1159/000511511. 33166961

[infa70109-bib-0002] Altmann, E. C. , M. Bazhydai , D. Karadağ , and G. Westermann . 2025. “The Infant and Toddler Curiosity Questionnaire: A Validated Caregiver‐Report Measure of Curiosity in Children From 5 to 24 Months.” Infancy 30, no. 1: e70001. 10.1111/infa.70001. 39853857 PMC11758190

[infa70109-bib-0003] Babik, I. , J. C. Galloway , and M. A. Lobo . 2022. “Early Exploration of One’s Own Body, Exploration of Objects, and Motor, Language, and Cognitive Development Relate Dynamically Across the First Two Years of Life.” Developmental Psychology 58, no. 2: 222–235. 10.1037/dev0001289. 34990201 PMC9589518

[infa70109-bib-0004] Bakeman, R. , and L. B. Adamson . 1984. “Coordinating Attention to People and Objects in Mother‐Infant and Peer‐Infant Interaction.” Child Development 55, no. 4: 1278. 10.2307/1129997. 6488956

[infa70109-bib-0005] Biro, P. , and J. Stamps . 2008. “Are Animal Personality Traits Linked to Life‐History Productivity?” Trends in Ecology & Evolution 23, no. 7: 361–368. 10.1016/j.tree.2008.04.003. 18501468

[infa70109-bib-0006] Blau, R. , and P. S. Klein . 2010. “Elicited Emotions and Cognitive Functioning in Preschool Children.” Early Child Development and Care 180, no. 8: 1041–1052. 10.1080/03004430802674316.

[infa70109-bib-0007] Bobak, C. A. , P. J. Barr , and A. J. O’Malley . 2018. “Estimation of an Inter‐Rater Intra‐Class Correlation Coefficient That Overcomes Common Assumption Violations in the Assessment of Health Measurement Scales.” BMC Medical Research Methodology 18, no. 1: 93. 10.1186/s12874-018-0550-6. 30208858 PMC6134634

[infa70109-bib-0008] Bornstein, M. H. , C. S. Hahn , and J. T. D. Suwalsky . 2013. “Physically Developed and Exploratory Young Infants Contribute to Their Own Long‐Term Academic Achievement.” Psychological Science 24, no. 10: 1906–1917. 10.1177/0956797613479974. 23964000 PMC4151610

[infa70109-bib-0009] Bourgeois, K. S. , A. W. Khawar , S. A. Neal , and J. J. Lockman . 2005. “Infant Manual Exploration of Objects, Surfaces, and Their Interrelations.” Infancy 8, no. 3: 233–252. 10.1207/s15327078in0803_3.

[infa70109-bib-0010] Bridgett, D. J. , M. A. Gartstein , S. P. Putnam , et al. 2011. “Emerging Effortful Control in Toddlerhood: The Role of Infant orienting/regulation, Maternal Effortful Control, and Maternal Time Spent in Caregiving Activities.” Infant Behavior and Development 34, no. 1: 189–199. 10.1016/j.infbeh.2010.12.008. 21186061

[infa70109-bib-0011] Bronfenbrenner, U. 1979. The Ecology of Human Development: Experiments by Nature and Design. Harvard University Press.

[infa70109-bib-0012] Bunford, N. , A. Kujawa , M. Dyson , T. Olino , and D. N. Klein . 2022. “Examination of Developmental Pathways From Preschool Temperament to Early Adolescent ADHD Symptoms Through Initial Responsiveness to Reward.” Development and Psychopathology 34, no. 3: 841–853. 10.1017/S0954579420002199. 33722319

[infa70109-bib-0013] Campos, J. J. , D. I. Anderson , M. A. Barbu‐Roth , E. M. Hubbard , M. J. Hertenstein , and D. Witherington . 2000. “Travel Broadens the Mind.” Infancy 1, no. 2: 149–219. 10.1207/S15327078IN0102_1. 32680291

[infa70109-bib-0014] Carey, W. B. 1981. “The Importance of Temperament‐Environment Interaction for Child Health and Development.” In The Uncommon Child, edited by M. Lewis and L. A. Rosenblum , 31–55. Springer US. 10.1007/978-1-4684-3773-7_3.

[infa70109-bib-0015] Colombo, J. , D. W. Mitchell , J. T. Coldren , and L. J. Freeseman . 1991. “Individual Differences in Infant Visual Attention: Are Short Lookers Faster Processors or Feature Processors?” Child Development 62, no. 6: 1247–1257. 10.2307/1130804. 1786713

[infa70109-bib-0016] Dalmaijer, E. S. , C. L. Nord , and D. E. Astle . 2022. “Statistical Power for Cluster Analysis.” BMC Bioinformatics 23, no. 1: 205–233. 10.1186/s12859-022-04675-1. 35641905 PMC9158113

[infa70109-bib-0017] Datavyu Team . 2014. “Datavyu: A Video Coding Tool.” [Computer Software] New York University. http://datavyu.org.

[infa70109-bib-0018] Degnan, K. A. , A. A. Hane , H. A. Henderson , O. L. Moas , B. C. Reeb‐Sutherland , and N. A. Fox . 2011. “Longitudinal Stability of Temperamental Exuberance and Social‐Emotional Outcomes in Early Childhood.” Developmental Psychology 47, no. 3: 765–780. 10.1037/a0021316. 21114347 PMC3984462

[infa70109-bib-0019] Dingemanse, N. J. , and P. de Goede . 2004. “The Relation Between Dominance and Exploratory Behavior Is Context‐Dependent in Wild Great Tits.” Behavioral Ecology 15, no. 6: 1023–1030. 10.1093/beheco/arh115.

[infa70109-bib-0020] Faul, F. , E. Erdfelder , A. Buchner , and A. Lang . 2009. “Statistical Power Analyses Using G * Power 3.1: Tests for Correlation and Regression Analyses.” Behavior Research Methods 41, no. 4: 1149–1160. 10.3758/BRM.41.4.1149. 19897823

[infa70109-bib-0021] Fidler, D. J. , E. Schworer , M. A. Prince , E. A. Will , A. W. Needham , and L. A. Daunhauer . 2019. “Exploratory Behavior and Developmental Skill Acquisition in Infants With Down Syndrome.” Infant Behavior and Development 54: 140–150. 10.1016/j.infbeh.2019.02.002. 30784761

[infa70109-bib-0022] Fox, N. A. , H. A. Henderson , K. H. Rubin , S. D. Calkins , and L. A. Schmidt . 2001. “Continuity and Discontinuity of Behavioral Inhibition and Exuberance: Psychophysiological and Behavioral Influences Across the First Four Years of Life.” Child Development 72, no. 1: 1–21. 10.1111/1467-8624.00262. 11280472

[infa70109-bib-0023] Franchak, J. M. 2020. “The Ecology of Infants’ perceptual‐motor Exploration.” Current Opinion in Psychology 32: 110–114. 10.1016/j.copsyc.2019.06.035. 31445428

[infa70109-bib-0024] Fredrickson, B. L. 1998. “What Good Are Positive Emotions?” Review of General Psychology 2, no. 3: 300–319. 10.1037/1089-2680.2.3.300. 21850154 PMC3156001

[infa70109-bib-0025] Fry, A. F. , and S. Hale . 1996. “Processing Speed, Working Memory, and Fluid Intelligence: Evidence for a Developmental Cascade.” Psychological Science 7, no. 4: 237–241. 10.1111/j.1467-9280.1996.tb00366.x.

[infa70109-bib-0026] Gartstein, M. A. , A. Prokasky , M. A. Bell , et al. 2017. “Latent Profile and Cluster Analysis of Infant Temperament: Comparisons Across Person‐Centered Approaches.” Developmental Psychology 53, no. 10: 1811–1825. 10.1037/dev0000382. 28758787 PMC5612890

[infa70109-bib-0027] Gartstein, M. A. , and M. K. Rothbart . 2003. “Studying Infant Temperament via the Revised Infant Behavior Questionnaire.” Infant Behavior and Development 26, no. 1: 64–86. 10.1016/S0163-6383(02)00169-8.

[infa70109-bib-0028] Gerhard, T. M. , J. C. Culham , and G. Schwarzer . 2021. “Manual Exploration of Objects Is Related to 7‐Month‐Old Infants’ Visual Preference for Real Objects.” Infant Behavior and Development 62: 101512. 10.1016/j.infbeh.2020.101512. 33310403

[infa70109-bib-0029] Gibson, E. J. 1988. “Exploratory Behavior in the Development of Perceiving, Acting, and the Acquirement of Knowledge.” Annual Review of Psychology 39, no. 1: 1–42. 10.1146/annurev.ps.39.020188.000245.

[infa70109-bib-0030] Gomez, R. , S. Watson , and A. Gomez . 2016. “Interrelationships of the Rothbart’s Temperament Model Constructs With Revised‐Reinforcement Sensitivity Theory Constructs.” Personality and Individual Differences 99: 118–121. 10.1016/j.paid.2016.04.072.

[infa70109-bib-0031] Hair, J. F. , W. C. Black , B. J. Babin , and R. E. Anderson . 2019. Multivariate Data Analysis. 8th ed. Cengage.

[infa70109-bib-0032] He, M. , E. A. Walle , and J. J. Campos . 2015. “A Cross‐National Investigation of the Relationship Between Infant Walking and Language Development.” Infancy 20, no. 3: 283–305. 10.1111/infa.12071.

[infa70109-bib-0033] Hervé, M. 2018. “RVAideMemoire: Testing and Plotting Procedures for Biostatistics.” [Computer Software] RVAideMemoire. 10.32614/CRAN.package.

[infa70109-bib-0034] Herzberg, O. , K. K. Fletcher , J. L. Schatz , K. E. Adolph , and C. S. Tamis‐LeMonda . 2022. “Infant Exuberant Object Play at Home: Immense Amounts of Time‐Distributed, Variable Practice.” Child Development 93, no. 1: 150–164. 10.1111/cdev.13669. 34515994 PMC8974536

[infa70109-bib-0035] Holland, C. M. , J. Sideris , B. L. Thompson , P. Levitt , and G. T. Baranek . 2023. “Exploring Development of Infant Gaze, Affect, and Object Exploration in a Primarily Latino Sample.” Infant Behavior and Development 70: 101806. 10.1016/j.infbeh.2022.101806. 36571914

[infa70109-bib-0036] Iverson, J. M. 2021. “Developmental Variability and Developmental Cascades: Lessons From Motor and Language Development in Infancy.” Current Directions in Psychological Science 30, no. 3: 228–235. 10.1177/0963721421993822. 34194130 PMC8240753

[infa70109-bib-0037] Kassambara, A. 2023. rstatix: Pipe‐Friendly Framework for Basic Statistical Tests. https://rpkgs.datanovia.com/rstatix/.

[infa70109-bib-0038] Klein, H. A. , and J. H. Ballantine . 1991. “Children’s Temperament: Patterns Across Cultures.” Journal of Research in Childhood Education 6, no. 1: 47–53. 10.1080/02568549109594821.

[infa70109-bib-0039] LeBarton, E. S. , and J. M. Iverson . 2013. “Fine Motor Skill Predicts Expressive Language in Infant Siblings of Children With Autism.” Developmental Science 16, no. 6: 815–827. 10.1111/desc.12069. 24118709 PMC3808875

[infa70109-bib-0040] Libertus, K. , A. S. Joh , and A. W. Needham . 2016. “Motor Training at 3 Months Affects Object Exploration 12 Months Later.” Developmental Science 19, no. 6: 1058–1066. 10.1111/desc.12370. 26689742 PMC4916043

[infa70109-bib-0041] Lilienfeld, S. O. 2010. “Can Psychology Become a Science?” Personality and Individual Differences 49, no. 4: 281–288. 10.1016/j.paid.2010.01.024.

[infa70109-bib-0042] Lobo, M. A. , and J. C. Galloway . 2013. “The Onset of Reaching Significantly Impacts How Infants Explore Both Objects and Their Bodies.” Infant Behavior and Development 36, no. 1: 14–24. 10.1016/j.infbeh.2012.09.003. 23261785

[infa70109-bib-0043] Lykken, D. T. 1991. “Thinking Clearly About Psychology.” In Essays on Matters of Public Interest, edited by D. Cicchetti and W. M. Grove . U of Minnesota Press.

[infa70109-bib-0044] Malachowski, L. G. , V. C. Salo , A. W. Needham , and K. L. Humphreys . 2023. “Infant Placement and Language Exposure in Daily Life.” Infant and Child Development 32, no. 3: e2405. 10.1002/icd.2405. 37694273 PMC10488907

[infa70109-bib-0045] Malachowski, L. G. , and A. W. Needham . 2023. “Infants Exploring Objects: A Cascades Perspective.” In Advances in Child Development and Behavior, edited by C. S. Tamis‐LeMonda and J. J. Lockman , Vol. 64, 39–68. 10.1016/bs.acdb.2022.11.001. 37080674

[infa70109-bib-0046] Masten, A. S. , and D. Cicchetti . 2010. “Developmental Cascades.” Development and Psychopathology 22, no. 3: 491–495. 10.1017/S0954579410000222. 20576173

[infa70109-bib-0047] Mlincek, M. M. , E. J. Roemer , C. Kraemer , and J. M. Iverson . 2022. “Posture Matters: Object Manipulation During the Transition to Arms‐Free Sitting in Infants at Elevated vs. Typical Likelihood for Autism Spectrum Disorder.” Physical & Occupational Therapy in Pediatrics 42, no. 4: 351–365. 10.1080/01942638.2022.2027845. 35086427 PMC9203937

[infa70109-bib-0048] Morales, S. , A. Tang , M. E. Bowers , et al. 2022. “Infant Temperament Prospectively Predicts General Psychopathology in Childhood.” Development and Psychopathology 34, no. 3: 774–783. 10.1017/S0954579420001996. 33432897 PMC8273182

[infa70109-bib-0049] Muentener, P. , E. Herrig , and L. Schulz . 2018. “The Efficiency of Infants’ Exploratory Play Is Related to Longer‐Term Cognitive Development.” Frontiers in Psychology 9: 635. 10.3389/fpsyg.2018.00635. 29904360 PMC5991261

[infa70109-bib-0050] Oakes, L. M. , and D. H. Rakison . 2019. Developmental Cascades: Building the Infant Mind. Oxford University Press: Incorporated. http://ebookcentral.proquest.com/lib/vand/detail.action?docID=5793970.

[infa70109-bib-0051] Oakes, L. M. , and D. J. Tellinghuisen . 1994. “Examining in Infancy: Does It Reflect Active Processing?” Developmental Psychology 30, no. 5: 748–756. 10.1037/0012-1649.30.5.748.

[infa70109-bib-0052] Palmer, C. F. 1989. “The Discriminating Nature of Infants’ Exploratory Actions.” Developmental Psychology 25, no. 6: 885–893. 10.1037/0012-1649.25.6.885.

[infa70109-bib-0053] Paterson, G. , and A. Sanson . 1999. “The Association of Behavioural Adjustment to Temperament, Parenting and Family Characteristics Among 5‐Year‐Old Children.” Social Development 8, no. 3: 293–309. 10.1111/1467-9507.00097.

[infa70109-bib-0054] Pfeifer, M. , H. H. Goldsmith , R. J. Davidson , and M. Rickman . 2002. “Continuity and Change in Inhibited and Uninhibited Children.” Child Development 73, no. 5: 1474–1485. 10.1111/1467-8624.00484. 12361313

[infa70109-bib-0055] Plomin, R. , and D. Daniels . 1984. “The Interaction Between Temperament and Environment: Methodological Considerations.” Merrill‐Palmer Quarterly 30, no. 2: 149–162. https://www.jstor.org/stable/23086230.

[infa70109-bib-0056] Posner, M. I. , and M. K. Rothbart . 2007. “Research on Attention Networks as a Model for the Integration of Psychological Science.” Annual Review of Psychology 58, no. 1: 1–23. 10.1146/annurev.psych.58.110405.085516. 17029565

[infa70109-bib-0057] Putnam, S. P. , A. L. Helbig , M. A. Gartstein , M. K. Rothbart , and E. Leerkes . 2014. “Development and Assessment of Short and Very Short Forms of the Infant Behavior Questionnaire–Revised.” Journal of Personality Assessment 96, no. 4: 445–458. 10.1080/00223891.2013.841171. 24206185

[infa70109-bib-0059] Quinn, J. L. , S. C. Patrick , S. Bouwhuis , T. A. Wilkin , and B. C. Sheldon . 2009. “Heterogeneous Selection on a Heritable Temperament Trait in a Variable Environment.” Journal of Animal Ecology 78, no. 6: 1203–1215. 10.1111/j.1365-2656.2009.01585.x. 19558612

[infa70109-bib-0060] Rachwani, J. , C. S. Tamis‐LeMonda , J. J. Lockman , L. B. Karasik , and K. E. Adolph . 2020. “Learning the Designed Actions of Everyday Objects.” Journal of Experimental Psychology: General 149, no. 1: 67–78. 10.1037/xge0000631. 31219298 PMC6923538

[infa70109-bib-0061] R Core Team . 2020. “R: A Language and Environment for Statistical Computing.” [Computer Software] R Foundation for Statistical Computing. https://www.r‐project.org/.

[infa70109-bib-0062] Réale, D. , S. M. Reader , D. Sol , P. T. McDougall , and N. J. Dingemanse . 2007. “Integrating Animal Temperament Within Ecology and Evolution.” Biological Reviews 82, no. 2: 291–318. 10.1111/j.1469-185X.2007.00010.x. 17437562

[infa70109-bib-0063] Revelle, W. 2022. “Psych: Procedures for Personality and Psychological Research (Version 2.2.5).”[Computer Software]. https://CRAN.R‐project.org/package=psych.

[infa70109-bib-0064] Rochat, P. 1989. “Object Manipulation and Exploration in 2‐ to 5‐Month‐Old Infants.” Developmental Psychology 25, no. 6: 871–884. 10.1037/0012-1649.25.6.871.

[infa70109-bib-0065] Rochat, P. , and N. Goubet . 1995. “Development of Sitting and Reaching in 5‐ to 6‐Month‐Old Infants.” Infant Behavior and Development 18, no. 1: 53–68. 10.1016/0163-6383(95)90007-1.

[infa70109-bib-0067] Rothbart, M. K. 1981. “Measurement of Temperament in Infancy.” Child Development 52, no. 2: 569. 10.2307/1129176.

[infa70109-bib-0068] Rothbart, M. K. 1988. “Temperament and the Development of Inhibited Approach.” Child Development 59, no. 5: 1241–1250. https://www.jstor.org/stable/1130487. 3168640

[infa70109-bib-0069] Rothbart, M. K. 2007. “Temperament, Development, and Personality.” Current Directions in Psychological Science 16, no. 4: 207–212. 10.1111/j.1467-8721.2007.00505.x.

[infa70109-bib-0070] Rothbart, M. K. , and J. E. Bates . 2006. “Temperament.” In Handbook of Child Psychology: Social, Emotional, and Personality Development. 6th ed., Vol. 3, 99–166. Wiley.

[infa70109-bib-0066] Rothbart, M. K. , B. E. Sheese , and E. D. Conradt . 2009. “Childhood Temperament.” In The Cambridge Handbook of Personality Psychology, edited by P. J. Corr and G. Matthews , 177–190. Cambridge University Press. 10.1017/CBO9780511596544.014.

[infa70109-bib-0071] Ruff, H. A. 1984. “Infants’ Manipulative Exploration of Objects: Effects of Age and Object Characteristics.” Developmental Psychology 20, no. 1: 9–20. 10.1037/0012-1649.20.1.9.

[infa70109-bib-0072] Ruff, H. A. , and C. J. Kohler . 1978. “Tactual—Visual Transfer in Six‐Month‐Old Infants.” Infant Behavior and Development 1: 259–264. 10.1016/S0163-6383(78)80037-X.

[infa70109-bib-0073] Ruff, H. A. , and M. K. Rothbart . 2001. Attention in Early Development: Themes and Variations. Oxford University Press.

[infa70109-bib-0074] Ruff, H. A. , L. M. Saltarelli , M. Capozzoli , and K. Dubiner . 1992. “The Differentiation of Activity in Infants’ Exploration of Objects.” Developmental Psychology 28, no. 5: 851–861. 10.1037/0012-1649.28.5.851.

[infa70109-bib-0075] Sameroff, A. 1975. “Transactional Models in Early Social Relations.” Human Development 18, no. 1–2: 65–79. 10.1159/000271476.

[infa70109-bib-0076] Schaffer, H. R. 1984. The Child’s Entry Into a Social World. Academic Press.

[infa70109-bib-0077] Schneider, J. L. , E. J. Roemer , J. B. Northrup , and J. M. Iverson . 2023. “Dynamics of the Dyad: How Mothers and Infants co‐construct Interaction Spaces During Object Play.” Developmental Science 26, no. 2: e13281. 10.1111/desc.13281. 35584243 PMC9840819

[infa70109-bib-0078] Seehagen, S. , C. Bartnick , J. Kärtner , et al. 2025. “Belong, Broaden, and Build: The Role of Positive Emotions in Early Human Development.” Child Development Perspectives 19, no. 4: 237–243. 10.1111/cdep.70002.

[infa70109-bib-0079] Soska, K. C. , and K. E. Adolph . 2014. “Postural Position Constrains Multimodal Object Exploration in Infants.” Infancy: The Official Journal of the International Society on Infant Studies 19, no. 2: 138–161. 10.1111/infa.12039. 24639621 PMC3951720

[infa70109-bib-0080] Squires, J. , and D. Bricker . 2009. Ages and Stages Questionnaire (ASQ): A Parent Completed Child Monitoring System. 3rd ed. Brooks Publishing Company.

[infa70109-bib-0081] Stifter, C. , M. Augustine , and J. Dollar . 2020. “The Role of Positive Emotions in Child Development: A Developmental Treatment of the Broaden and Build Theory.” Journal of Positive Psychology 15, no. 1: 89–94. 10.1080/17439760.2019.1695877.

[infa70109-bib-0082] Stifter, C. , S. Putnam , and L. Jahromi . 2008. “Exuberant and Inhibited Toddlers: Stability of Temperament and Risk for Problem Behavior.” Development and Psychopathology 20, no. 2: 401–421. 10.1017/S0954579408000199. 18423086 PMC3732742

[infa70109-bib-0083] Super, C. M. , G. Axia , S. Harkness , et al. 2008. “Culture, Temperament, and the ‘Difficult Child’: A Study in Seven Western Cultures.” International Journal of Developmental Science 2, no. 1–2: 136–157. 10.3233/DEV-2008-21209.

[infa70109-bib-0084] Tamis‐LeMonda, C. S. , Y. Kuchirko , and L. Tafuro . 2013. “From Action to Interaction: Infant Object Exploration and Mothers’ Contingent Responsiveness.” IEEE Transactions on Autonomous Mental Development 5, no. 3: 202–209. 10.1109/TAMD.2013.2269905.

[infa70109-bib-0085] Taylor, M. A. , S. Coxe , and E. L. Nelson . 2024. “Early Object Skill Supports Growth in Role‐Differentiated Bimanual Manipulation in Infants.” Infant Behavior and Development 74: 101925. 10.1016/j.infbeh.2024.101925. 38286042 PMC11194832

[infa70109-bib-0086] Thomas, A. , and S. Chess . 1977. Temperament and Development. Brunner/Mazel. https://psycnet.apa.org/Record/1978‐03178‐000.

[infa70109-bib-0087] Thurman, S. L. , and D. Corbetta . 2017. “Spatial Exploration and Changes in Infant–Mother Dyads Around Transitions in Infant Locomotion.” Developmental Psychology 53, no. 7: 1207–1221. 10.1037/dev0000328. 28459258

[infa70109-bib-0088] U.S. Department of Housing and Urban Development . 2020. “FY 2020 Income Limits Documentation System.” https://www.huduser.gov/portal/datasets/il.html#year2020.

[infa70109-bib-0089] Von Hofsten, C. 1982. “Eye–Hand Coordination in the Newborn.” Developmental Psychology 18, no. 3: 450–461. 10.1037/0012-1649.18.3.450.

[infa70109-bib-0090] Wang, M. V. , R. Lekhal , L. E. Aarø , and S. Schjølberg . 2014. “Co‐Occurring Development of Early Childhood Communication and Motor Skills: Results From a Population‐Based Longitudinal Study.” Child: Care, Health and Development 40, no. 1: 77–84. 10.1111/cch.12003. 22970997

[infa70109-bib-0091] Wolff, A. 2020. “Chapter 2—Hand Function: Typical Development.” In Pediatric Hand Therapy, edited by J. M. Abzug , S. H. Kozin , and R. Neiduski , 13–23. Elsevier. 10.1016/B978-0-323-53091-0.00002-6.

[infa70109-bib-0092] Yu, C. , and L. B. Smith . 2016. “The Social Origins of Sustained Attention in One‐Year‐Old Human Infants.” Current Biology 26, no. 9: 1235–1240. 10.1016/j.cub.2016.03.026. 27133869 PMC5387765

[infa70109-bib-0093] Yu, C. , S. H. Suanda , and L. B. Smith . 2019. “Infant Sustained Attention but Not Joint Attention to Objects at 9 Months Predicts Vocabulary at 12 and 15 Months.” Developmental Science 22, no. 1: e12735. 10.1111/desc.12735. 30255968 PMC6918481

[infa70109-bib-0094] Zeanah, C. H. , and P. D. Zeanah . 2019. In Handbook of Infant Mental Health, edited by C. H. Zeanah . 4th ed. Guilford Press.

[infa70109-bib-0095] Zentner, M. , and R. L. Shiner , eds. 2012. Handbook of Temperament. Guilford Press.

[infa70109-bib-0096] Zuccarini, M. , A. Guarini , J. M. Iverson , et al. 2018. “Does Early Object Exploration Support Gesture and Language Development in Extremely Preterm Infants and Full‐Term Infants?” Journal of Communication Disorders 76: 91–100. 10.1016/j.jcomdis.2018.09.004. 30300842

[infa70109-bib-0097] Zuccarini, M. , A. Guarini , S. Savini , et al. 2017. “Object Exploration in Extremely Preterm Infants Between 6 and 9 Months and Relation to Cognitive and Language Development at 24 Months.” Research in Developmental Disabilities 68: 140–152. 10.1016/j.ridd.2017.06.002. 28779627

