# Supplementary material for: Understanding Object Exploration: The Role of Temperament Early in Life
Source: Infancy. 2026 Jul 15;31(4):e70109. doi: 10.1111/infa.70109 (PMC13373247; doi:10.1111/infa.70109)
Supplement: Supplementary file 1 — Supporting Information S1 [file INFA-31-0-s001.docx]

**Supplementary Materials for**

***Understanding Object Exploration: The Role of Temperament Early in Life***

**Overview**

We present the following information in the Supplementary Materials, in the order that it is

referenced in the main manuscript:

1. Figure S1. Boxplot Depicting Fine Motor Exploration by Cluster
2. Table S1. Mean Values of Temperament Factors by Cluster

**Figure S1**

*Boxplot Depicting Fine Motor Exploration by Cluster*

*
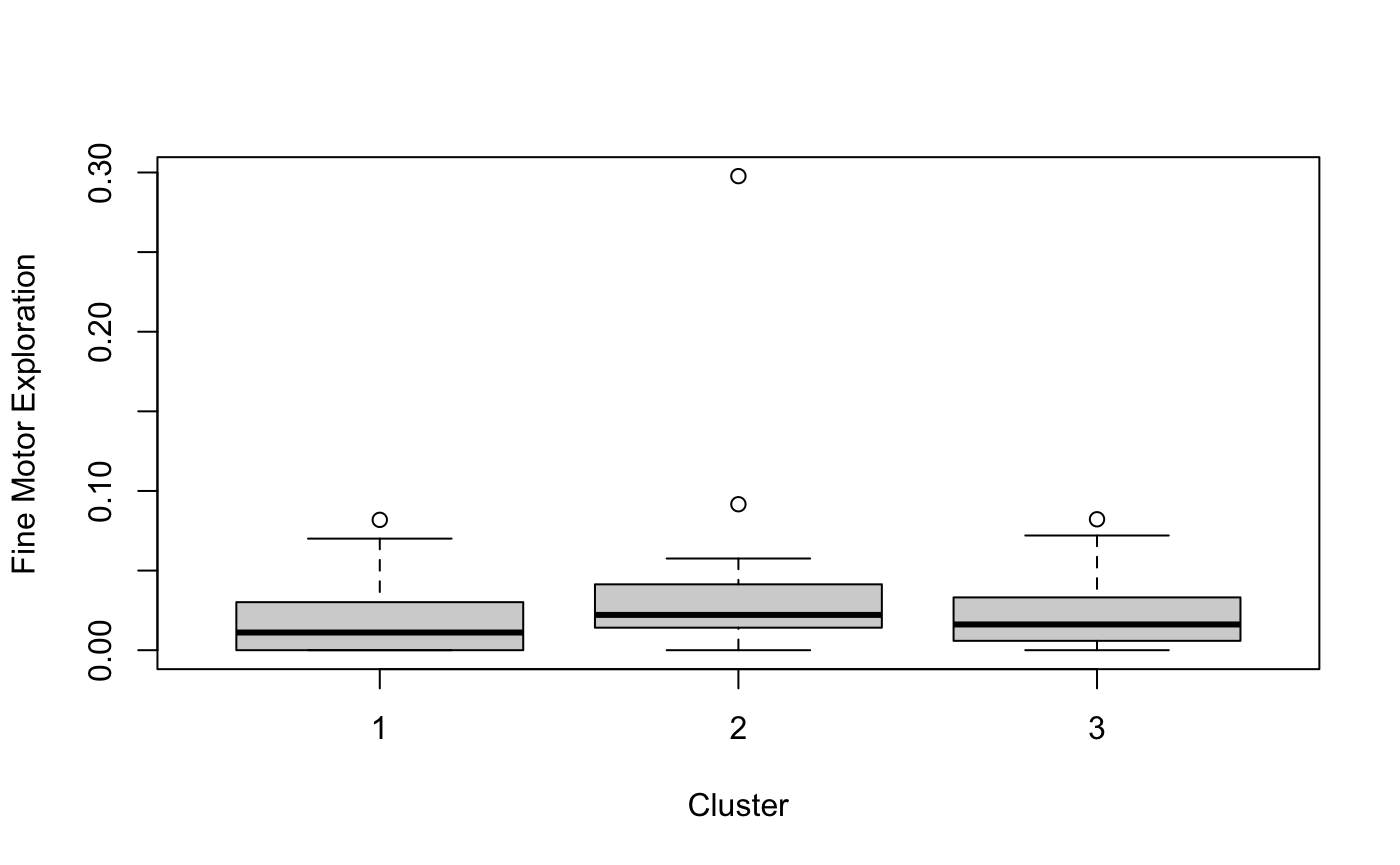
*

*Note.* Fine motor exploration was analyzed as a proportion of total codable time in the free play task video (e.g., a score of .05 indicates that fine motor exploration occurred for 5% of the codable time in the video).

**Table S1**

*Mean Values of Temperament Factors by Cluster*

|  | Cluster 1  (*n* = 37) | Cluster 2  (*n* = 28) | Cluster 3  (*n* = 31) |
| --- | --- | --- | --- |
| Activity Level (*M* = 4.39, *SD* = 0.96) | 4.40 | 5.06 | 3.76 |
| Approach (*M* = 5.57, *SD* = 0.72) | 5.73 | 5.81 | 5.14 |
| Vocalization (*M* = 4.86, *SD* = 1.01) | 5.44 | 5.19 | 3.89 |
| Smiling (*M* = 4.58, *SD* = 1.07) | 5.29 | 4.72 | 3.61 |
| High Intensity Pleasure (*M* = 5.76, *SD* = 0.74) | 6.10 | 5.93 | 5.20 |
| Low Intensity Pleasure (*M* = 5.52, *SD* = 0.89) | 6.04 | 5.48 | 4.93 |
| Soothability (*M* = 5.77, *SD* = 0.81) | 6.32 | 5.25 | 5.58 |
| Sadness (*M* = 3.52, *SD* = 0.97) | 3.00 | 4.16 | 3.56 |
| Distress to Limitations (*M* = 3.69, *SD* = 0.93) | 3.17 | 4.58 | 3.50 |
| Fear (*M* = 2.68, *SD* = 1.15) | 2.29 | 3.47 | 2.43 |
| Cuddliness (*M* = 5.94, *SD* = 0.64) | 6.12 | 5.76 | 5.90 |
| Perceptual Sensitivity (*M* = 4.23, *SD* = 1.45) | 4.59 | 4.60 | 3.48 |
| Falling Reactivity (*M* = 2.85, *SD* = 0.87) | 2.30 | 3.60 | 2.82 |
| Duration of Orienting (*M* = 3.96, *SD* = 1.14) | 4.59 | 4.06 | 3.13 |

*Note*. Clusters were identified using *k*-means cluster analysis based on scores on each of the 14 individual IBQ dimensions.
